# Optimal duration between drainage for obstructing renal or ureteral stones associated with infection and ureteroscopic lithotripsy: a randomized controlled trial

**DOI:** 10.1007/s00345-025-06124-z

**Published:** 2026-03-10

**Authors:** Mahmoud E. Helal, Ahmed R. EL-Nahas, Mahmoud Laymon, Amr A. Elsawy, Yasser Osman

**Affiliations:** Urology Department, Urology and Nephrology Center, Mansoura University, Mansoura, 35516 Egypt

**Keywords:** Ureteroscopy, Urosepsis, Urinary calculi, Ureteral stent, UTI

## Abstract

**Objective:**

This study was done to determine the optimal interval between drainage of obstructed infected kidney and ureteroscopic lithotripsy.

**Patients and methods:**

This randomized controlled trial was conducted between May 2023 and July 2024. Inclusion criteria were adult patients with obstructed infected kidneys by renal or ureteric stones. After drainage of the kidney, they were randomized into early and delayed groups. In the early group, ureteroscopy was done after 7 days while in the delayed group, ureteroscopy was done after 14–21 days of drainage. The primary outcome was the difference in incidence of postoperative infectious complications. Infectious complications were defined as having one of the criteria of systemic inflammatory response syndrome (SIRS) with infected urine culture. Secondary outcomes were unplanned visits during the waiting period, hospital stay and stone-free rates (SFR).

**Results:**

The study included 96 patients (44 in early group and 52 in delayed group). Post-ureteroscopy infectious complications were significantly more in the early group (56.8% versus 30.8%, P = 0.01). The rate of unplanned hospital visits during waiting period was higher in the delayed group (36.5% versus 11.4% in the early group, P = 0.005). Hospital stays after ureteroscopy and SFR were comparable between both groups (P = 0.227 and P = 0.464).

**Conclusions:**

The preferred timing for ureteroscopy after drainage of obstructed infected kidneys with urolithiasis may be between 14 to 21 days because early intervention was associated with a higher incidence of infectious complications.

**Trial registration:**

The protocol for the trial was registered in ClinicalTrials.gov (NCT06101563) on October 20, 2023.

## Introduction

Urolithiasis is a growing health problem with an increasing prevalence reaching 9%-11% [1]**.** A subset of patients with obstructing upper tract calculi presents with infection, requiring urgent renal decompression to prevent morbidity and mortality [2]. Drainage is achieved via retrograde ureteral stenting or placement of a percutaneous nephrostomy (PCN) tube [3]**.** Following decompression and infection control, patients can undergo definitive stone management [4].

Despite its importance, no consensus exists on the optimal timing of definitive endoscopic intervention following drainage of obstructed, infected kidney [5]. In practice, the decision is influenced by urologist experience and hospital policy. Delaying intervention may allow more complete infection clearance, but also prolongs exposure to stent related morbidity and increases healthcare burden [6–8]**.** Extended antibiotic use during this period adds risks of adverse effects and resistant organisms [9].

Urologists should therefore balance adequate infection control with the complications of prolonged drainage, antibiotic use, and reduced quality of life. This study aims to define the optimal interval between drainage and ureteroscopic lithotripsy in patients with infection-associated obstructing stones.

## Patients and methods

This prospective randomized trial was approved by the local ethics committee (MS.23.04.2371) and prospectively registered at ClinicalTrials.gov (Identifier: NCT 06101563). Written informed consent was obtained from all participants prior to enrollment, in accordance with the Declaration of Helsinki.

Eligibility criteria included patients aged ≥ 18 years presenting with an obstructing ureteral or renal stone ≤ 20 mm and concomitant upper urinary tract infection meeting criteria for systemic inflammatory response syndrome (SIRS). Exclusion criteria were prior urinary tract instrumentation within four weeks, bilateral obstruction, **no response to drainage and antibiotics,** or associated emphysematous pyelonephritis/perinephric abscess.

### Initial drainage

All patients received intravenous third-generation cephalosporins at presentation, followed by decompression of the obstructed system either by ureteral stent (6F/24 or 6F/26 double-pigtail, Vortek®, Coloplast, Humlebæk, Denmark) or percutaneous nephrostomy. The choice of drainage method was determined jointly by the anesthesiologist and urologist. After drainage, patients were hospitalized until normalization of temperature and leukocyte count. Antibiotic therapy was subsequently adjusted based on urine culture and sensitivity results for 7 days.

### Randomization and study groups

Participants were randomized in a 1:1 ratio using computer-generated randomization tables into two groups. Group E (Early) underwent ureteroscopic lithotripsy 7 days after drainage, whereas Group D (Delayed) underwent the procedure 14–21 days after drainage. Allocation was concealed by means of sequentially numbered, opaque, sealed envelopes prepared by an independent research assistant not involved in patient recruitment or outcome assessment. The envelopes were opened only after patient enrollment and consent, thereby ensuring allocation concealment. The operating surgeon was aware of group allocation, whereas outcome assessors were blinded to group assignment.

### Re-evaluation before ureteroscopic treatment

During the waiting period between drainage and definitive treatment, all patients were assessed for unplanned visit or hospital readmissions. Re-evaluation protocol included assessment of the infection indices (body temperature, leukocytic count, urine analysis and culture). Patients with infected urine cultures were treated prior to definitive intervention **with culture-specific antibiotics for another 5 days**. Patient-reported QoL was assessed using the Arabic version of the Short Form 36-Item Survey (https://www.rand.org/health/surveys_tools/mos/36-item-short-form.html).

### Definitive treatment

A semirigid ureteroscope (Richard Wolf GmbH, Knittlingen, Germany) was used for distal ureteral stones. A single-use digital flexible ureteroscope (LithoVue™, Boston scientific, Massachusetts, USA) was used for proximal ureteral or renal stones A ureteral access sheath (11/13 F, Navigator™, Boston Scientific, USA) was routinely employed. Laser lithotripsy (Cyber Ho 150 Watt, Quanta Systems, Italy) was performed using either dusting or fragmentation at the surgeon’s discretion. At the end of the procedure, drainage method was 6 F external ureteral catheter for one or two days. At the conclusion of the procedure, external ureteral catheters (6F) were placed for 1–2 days. Ureteral stents (DJ) were used in cases of residual fragments, intraoperative complications (bleeding or ureteric injury), or marked ureteral edema, and were removed after four weeks.

### Follow–up and outcomes

All patients were monitored during the postoperative period for pain and postoperative complications which were graded using the modified Clavien system [10].

**Primary outcome:** incidence of post-URS infectious complications between study groups. We used SIRS criteria (temperature > 38.3 °C or < 36 °C, heart rate > 90/min, respiratory rate > 20/min, or WBC > 12,000/mm^3^ or < 4,000/mm^3^) to identify patients with systemic inflammatory response in the setting of obstructed, infected upper tract stones. Sepsis was diagnosed when ≥ 2 criteria were present.

**Secondary outcomes:** included rate and causes of unplanned visits and hospital readmissions during the waiting periods, patient-reported QoL during the waiting period, rate, grade and type of post-URS complications, post-URS length of hospital stay, and SFR that was assessed in all patients after four weeks by NCCT.

### Sample size calculation

Previous data demonstrated a significantly higher occurrence of postoperative SIRS when ureteroscopy was performed within 14 days (38.8%) compared with ≥ 14 days (12.5%), corresponding to an absolute difference of ~ 26% [14]. Based on this, we assumed a 30% absolute difference in the incidence of infectious complications between early and delayed intervention, which we considered clinically meaningful. Using a two-sided α = 0.05 and 80% power for a test of two independent proportions, the required sample size was calculated to be 38 patients per group. To account for a potential 10% attrition rate, we targeted 44 participants per group. The final analyzed cohorts included 44 patients in the early group and 52 in the delayed group.

### Statistical analysis

Data analysis was conducted using SPSS v21 (Chicago, Ill, USA). Chi-square and Fisher exact tests were used to compare categorical variables while independent simple t-test or Mann–Whitney U-test were used as appropriate to compare continuous variables. Binary logistic regression was used for multivariate analysis. A critical two-sided P-value < 0.05 was used for statistically significant differences.

## Results

### Patients flow

**Fig. 1:**
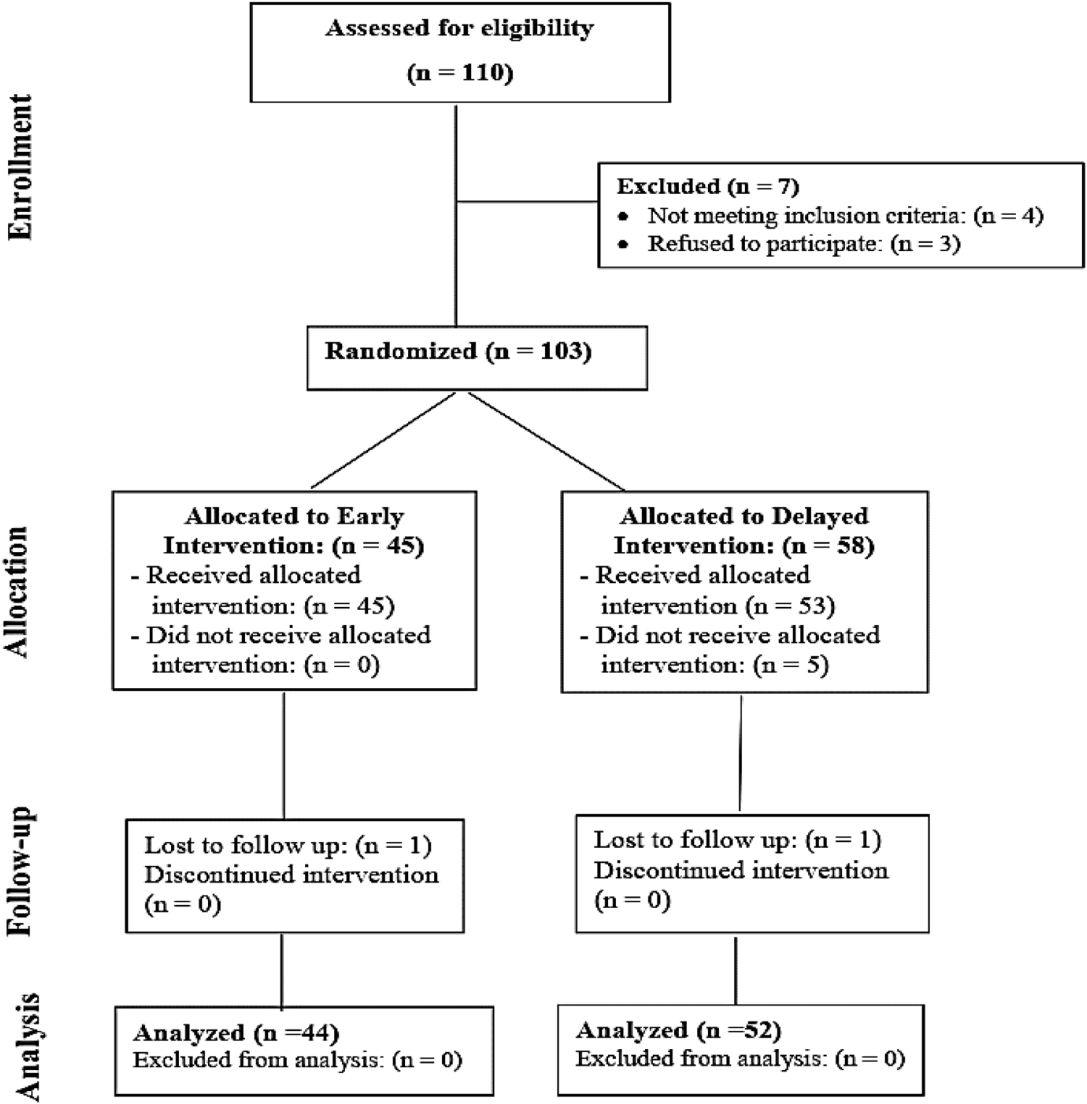
CONSORT chart for patients’ flow through the study phases

Patients’ flow through the study phasis is shown in Fig. 1. Of 103 randomized, six in the delayed group were excluded: three passed stones before intervention, two were lost after drainage, and one after surgery. In the early group, one was lost after intervention. Thus, statistical analysis included 44 patients in group E and 52 in group D.

### Preoperative data

Preoperative patients’, renal and stone characters in both groups are summarized in Table 1. Eighty-five patients presented with high-grade fever (> 38.5° Celsius). Seven patients required intensive care unit admission for management of septic shock. All patients underwent renal drainage with ureteric stent except three who required PCN.

**Table 1:** Preoperative Patients and Renal Characters for Treatment Groups

Parameters	Early Group 44 Patients	Delayed Group 52 patients	P Value
	Number (%)	Number (%)	
Age (years)	46.4 ±13.6	49.2 ±15.8	0.331^&^
BMI (kg/m^2^)	31.8 ±7.4	31.9 ±7.1	0.940^&^
Serum Creatinine (mg/dl)	1.95 ±1.44	2.14±1.94	0.590^&^
Gender			0.572^*^
Male	22 (50)	23 (44.2)	
Female	22 (50)	29 (55.8)	
Previous Stone Treatment			0.553^*^
No	28 (63.6)	30 (57.7)	
Yes	16 (36.4)	22 (42.3)	
Diabetes			0.464^*^
No	38 (86.4)	42 (80.8)	
Yes	6 (13.6)	10 (19.2)	
Hypertension			0.803^*^
No	29 (65.9)	33 (63.5)	
Yes	15 (34.1)	19 (36.5)	
Hydronephrosis			0.809^*^
Mild	38 (86.4)	44 (84.6)	
Moderate	6 (13.6)	8 (15.4)	
Urine Culture:			0.707^*^
Sterile	12 (27.3)	16 (30.8)	
Infected	32 (72.7)	36 (69.2)	
Stone Side			0.782^*^
Right	25 (56.8)	31 (59.6)	
Left	19 (43.2)	21 (40.4)	
Stone Site			0.147^*^
Ureteropelvic Junction	2 (4.5)	9 (17.3)	
Upper Ureter	23 (52.3)	23 (44.2)	
Lower Ureter	19 (43.2)	20 (38.5)	
Stone Multiplicity			0.143^*^
Single	34 (77.3)	46 (88.5)	
Multiple	10 (22.7)	6 (11.5)	
Stone Length (mm) (mean ± SD)	8.2 (3.5)	9.5 (4.7)	0.140^&^
Stone Density (HU) (mean ± SD)	763.3 (333.3)	780.6 (338.6)	0.808^&^

*Chi-square test

^&^Independent sample t-test

### Waiting period data

The mean pain analogue score (PAS) was comparable between both groups (2.6 ± 1.1 and 2.5 ± 1.3 for group E and D respectively, P = 0.773). As were QoL scores (48.2 ± 10.6 vs. 49.4 ± 10.8; p = 0.593). However, unplanned hospital visits were significantly more frequent in the delayed group (36.5% vs. 11.4%; p = 0.005), largely attributed to stent-related lower urinary tract symptoms. Hospital readmissions were comparable (15.4% in group D vs. 11.4% in group E; p = 0.556), mostly for acute pyelonephritis requiring catheter drainage and culture-directed antibiotics. One patient in group D required stent exchange for distal migration. The remaining 11 unplanned visits in group D were due to storage LUTS, which were managed conservatively with anticholinergic medications. **Urine cultures after drainage were infected in 32 patients (72.7%) in group E and 36 patients (69.2%) in group D, P =0. 707**.

### Postoperative data

The incidence and severity of postoperative infectious complications were significantly more in group E (56.8%) versus 30.8% in group D (95%CI 1.14–2.99, P = 0.010) (Table 2). Postoperative complications were managed with analgesics and antipyretics for pain or fever. If the patient developed sepsis, IV antibiotics and fluids were added to the treatment. Septic shock occurred in one patient in the delayed group and was managed in the ICU for monitoring the vital signs with addition of IV fluids, antibiotics and vasopressors. The median hospital stay was comparable between both groups (2 days, range (1–6) in group E vs. 1 day range (1–6) in group D; P = 0.227).

**Table 2:** Postoperative Data for Treatment Groups:

Parameters	Early Group 44 Patients	Delayed Group 52 patients	P value *
	Number (%)	Number (%)	
Infectious Complications			0.010
No	19 (43.2)	36 (69.2)	
Yes	25 (56.8)	16 (30.8)	
Leukocytosis (> 12,000 cells/mm^3^)			0.019
No	21 (47.7)	37 (71.2)	
Yes	23 (52.3)	15 (28.8)	
Fever: (> 38 °C)			0.355
No	33 (75)	43 (82.7)	
Yes	11 (25)	9 (17.3)	
Sepsis			0.358
No	34 (77.3)	44 (84.6)	
Yes	10 (27.7)	8 (15.4)	
Modified Clavien Classification			0.035
No	19 (43.2)	36 (69.2)	
G1	12 (27.3)	7 (13.5)	
G2	13 (29.5)	9 (17.3)	
Stone-Free			0.464
Yes	38 (86.4)	42 (80.8)	
Residual stones	6 (13.6)	10 (19.2)	

*Chi-square test

### Risk factor analysis

On univariate analysis, baseline factors, including gender, stone laterality, diabetes, prior stone treatment and urine culture were not significantly associated with infectious complications (all P > 0.5). Patients with infectious complications had significantly higher leukocytic counts at presentation (median 10.7 vs. 9.1 × 10^3^/mm^3^; P = 0.028), and a trend toward higher risk was observed in those with moderate hydronephrosis (22% vs. 9.1%; P = 0.077). Early definitive intervention was associated with a markedly higher incidence of infectious complications compared with delayed intervention (61% vs. 34.5%; P = 0.010) Table 3.

**Table 3:** Univariate Analysis for Risk Factors of Infectious Complications

Parameters	Infectious Complications		P Value
	No (55 Patients)	Yes (41 patients)	
	Number (%)	Number (%)	
Age (years) (mean ± SD)	48.1 ± 16.3	47.4 ± 12.8	0.831^&^
Leukocytic count (X1000/mm^3^)	9.1 ± 3.2	10.7 ± 3.9	0.028^&^
Gender			0.928^*^
Male	26 (47.3)	19 (46.3)	
Female	29 (52.7)	22 (53.7)	
Side			0.972^*^
Right	32 (58.2)	24 (58.5)	
Left	23 (41.8)	17 (41.5)	
Diabetes			0.310^*^
No	44 (80)	38 (87.8)	
Yes	11 (20)	15 (36.6)	
Previous Stone Treatment			0.604^*^
No	32 (58.2)	26 (63.4)	
Yes	23 (41.8)	22 (42.3)	
Preoperative Urine Culture			0.985^*^
Sterile	16 (29.1)	12 (29.3)	
Infected	39 (70.9)	29 (70.7)	
Hydronephrosis			0.077^*^
Mild	50 (90.9)	32 (78)	
Moderate	5 (9.1)	9 (22)	
Group			0.010^*^
Early Intervention (E)	19 (34.5)	25 (61)	
Delayed Intervention (D)	36 (65.5)	16 (39)	
Stone Length (mm) (mean ± SD)	9.4 ± 4.6	8.3 ± 3.6	0.229^&^
Stone Density (HU) (mean ± SD)	744 ± 341	811 ± 352	0.350^&^

^*^Chi-square test

^&^Independent sample t-test

In the multivariate logistic regression, only the timing of intervention remained an independent predictor, with early intervention conferring nearly a threefold higher risk (OR 2.96, 95%CI 1.28–6.85; P = 0.011). Neither leukocytosis nor hydronephrosis retained statistical significance after adjustment, suggesting that these factors may be confounded by or mediated through timing of surgery.

## Discussion

Obstructing urolithiasis with urinary tract infection is a serious emergency, with urosepsis being the leading cause of stone-related mortality [11]. Immediate drainage is essential, yet the optimal timing of definitive stone treatment remains controversial, with no guideline consensus.

Prior studies report conflicting results. Shi et al. [12] found ureteroscopy at a mean of 8 days post-drainage safe, with postoperative UTI rates of 6%, comparable to treatment at 13 days. Orr et al. [13] also found no effect of timing on postoperative urosepsis. In contrast, Yoo et al. [14] showed that antibiotic courses shorter than 14 days were linked to higher postoperative SIRS rates. These discrepancies reflect heterogeneity in study populations, timing, and inclusion criteria.

Our study focused on patients with recent obstructive pyelonephritis, a high-risk cohort rarely isolated in prior work. Ureteroscopy within 7 days of drainage carried a nearly threefold higher risk of infectious complications (56.8% vs. 30.8%; OR 2.9, 95% CI 1.28–6.85, p = 0.010). Early intervention was the only independent predictor in multivariate analysis. This indicates that even after clinical resolution and sterile cultures, residual parenchymal inflammation or subclinical bacterial persistence may trigger infection if intervention is premature. Culture negativity also does not exclude biofilm or intracellular bacteria that may reactivate during URS.

In our early group, sepsis incidence was 27.7%, far higher than the 8–12% reported in unselected ureteroscopy cohorts, highlighting recent obstructive pyelonephritis as a distinct high-risk subgroup [15, 16]. Our findings align with Itami et al., who reported febrile UTI in 53% of patients with prior sepsis versus 33% without [17]. Although postoperative sepsis was numerically higher in the early group (27.7% vs. 15.4%), this did not reach significance, likely due to limited sample size. Still, both rates were markedly above the < 1% reported in general ureteroscopy series [18, 19], reinforcing the elevated risk in this population.

Multivariate analysis identified early ureteroscopy as the only independent risk factor for infectious complications, supporting a strategy of waiting at least two weeks before definitive treatment. Other suspected predictors—including diabetes, stone size, hydronephrosis, and preoperative urine culture—were not significant. The lack of association with culture likely reflects routine preoperative antibiotics, which sterilize bladder urine but not renal parenchyma or stent biofilms.

Conversely, delaying beyond 3–4 weeks also carries risks. Itami et al. showed that stent dwell time > 21 days independently increased febrile UTI, attributed to progressive colonization rising from minimal at 2 weeks to > 70% at 6 weeks [20]. In our cohort, delayed URS caused more unplanned visits for stent-related symptoms, though readmission rates were similar. Quality of life impairment was also comparable, consistent with prior studies showing that stents invariably reduce QoL and longer dwell times worsen outcomes [21–23]. Thus, balancing infection risk against stent morbidity is crucial.

Overall, our findings support a narrow therapeutic window where intervention should be avoided before 14 days, when infection risk is high, and beyond 21–28 days, when stent morbidity increases. The 14–21 days interval appears optimal for definitive URS in patients recovering from obstructive pyelonephritis.

Our study has some limitations. The single-center design and relatively short follow-up (limited to four weeks) restrict the generalizability and long-term interpretation of our findings. In addition, the modest sample size may have underpowered some subgroup analyses, such as the risk of sepsis. Although drainage methods were not fully uniform, ureteral stenting predominated (93.8%), minimizing potential heterogeneity. We used SIRS criteria for its high sensitivity in emergency settings [24]. This approach aligns with prior study in obstructive pyelonephritis [15]. Future multicenter studies are warranted to validate these results, explore biomarker-based risk stratification (e.g., procalcitonin, CRP), and better define the optimal timing window for safe ureteroscopy following obstructive pyelonephritis.

## Conclusions

**Based on the results of this study,** the preferred timing for ureteroscopy after drainage of obstructed infected kidneys with urolithiasis may be between 14 to 21 days because early intervention was associated with a higher incidence of infectious complications.

## Data Availability

The data is available on request.

## Declarations

content of this article.
